# Hominin Footprints from Early Pleistocene Deposits at Happisburgh, UK

**DOI:** 10.1371/journal.pone.0088329

**Published:** 2014-02-07

**Authors:** Nick Ashton, Simon G. Lewis, Isabelle De Groote, Sarah M. Duffy, Martin Bates, Richard Bates, Peter Hoare, Mark Lewis, Simon A. Parfitt, Sylvia Peglar, Craig Williams, Chris Stringer

**Affiliations:** 1 Department of Prehistory and Europe, British Museum, London, United Kingdom; 2 Institute of Archaeology, University College London, London, United Kingdom; 3 School of Geography, Queen Mary University of London, London, United Kingdom; 4 Research Centre in Evolutionary Anthropology and Palaeoecology, Liverpool John Moores University, Liverpool, United Kingdom; 5 Department of Archaeology, University of York, York, United Kingdom; 6 Department of Archaeology, University of Wales Trinity St David, Lampeter, United Kingdom; 7 School of Geography and Geosciences, University of Andrews, St Andrews, United Kingdom; 8 Department of Geology, Norwich Castle Museum & Art Gallery, Norwich, United Kingdom; 9 Department of Earth Sciences, Natural History Museum, London, United Kingdom; 10 School of Botany, University of Cambridge, Cambridge, United Kingdom; University of Oxford, United Kingdom

## Abstract

Investigations at Happisburgh, UK, have revealed the oldest known hominin footprint surface outside Africa at between ca. 1 million and 0.78 million years ago. The site has long been recognised for the preservation of sediments containing Early Pleistocene fauna and flora, but since 2005 has also yielded humanly made flint artefacts, extending the record of human occupation of northern Europe by at least 350,000 years. The sediments consist of sands, gravels and laminated silts laid down by a large river within the upper reaches of its estuary. In May 2013 extensive areas of the laminated sediments were exposed on the foreshore. On the surface of one of the laminated silt horizons a series of hollows was revealed in an area of ca. 12 m^2^. The surface was recorded using multi-image photogrammetry which showed that the hollows are distinctly elongated and the majority fall within the range of juvenile to adult hominin foot sizes. In many cases the arch and front/back of the foot can be identified and in one case the impression of toes can be seen. Using foot length to stature ratios, the hominins are estimated to have been between ca. 0.93 and 1.73 m in height, suggestive of a group of mixed ages. The orientation of the prints indicates movement in a southerly direction on mud-flats along the river edge. Early Pleistocene human fossils are extremely rare in Europe, with no evidence from the UK. The only known species in western Europe of a similar age is *Homo antecessor*, whose fossil remains have been found at Atapuerca, Spain. The foot sizes and estimated stature of the hominins from Happisburgh fall within the range derived from the fossil evidence of *Homo antecessor*.

## Introduction

**Figure 1:**
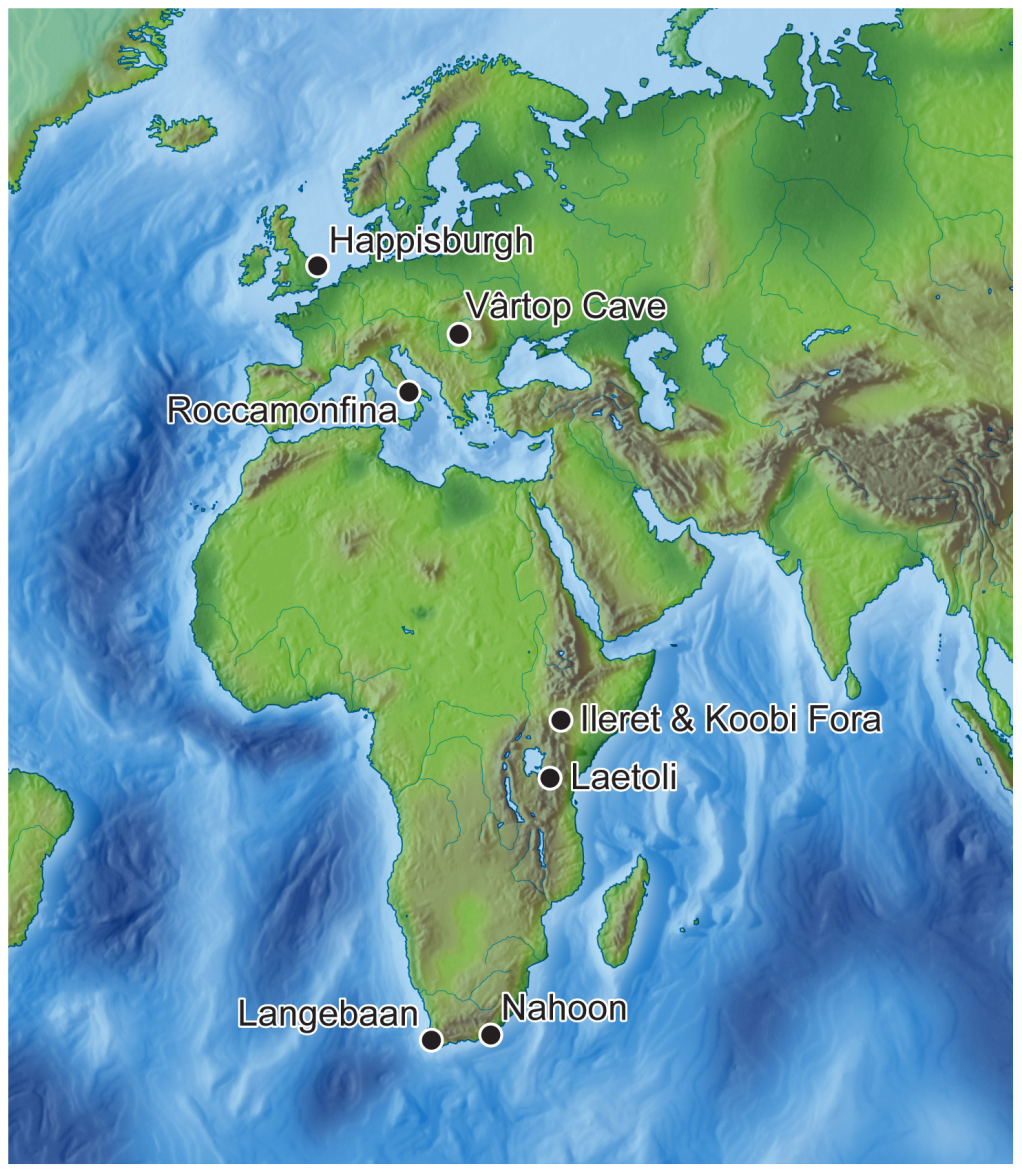
Map of Pleistocene footprint sites dating from prior to 40 ky in Africa and Eurasia.

The survival of early hominin footprints is extremely rare, but can provide critical information about bipedalism, posture, gait and the size of the hominins involved, often in the absence of skeletal evidence [1]–[8] (Figure 1). In cases where multiple footprints are preserved, the number of individuals, the sex and age range of the group and the activities can be inferred [4], [7], [9]–[10]. The earliest hominin footprints are preserved in volcanic ash at Laetoli in Tanzania. These provide evidence of bipedalism in *Australopithecus afarensis* dating to ca. 3.66 million years ago (My) [1], [11]–[13]. In the Early Pleistocene at ca. 1.5 My two sites have been discovered just to the east of Lake Turkana (Kenya) with footprints of *Homo erectus* or possibly *Paranthropus boisei*. The first site is in sediments of the Koobi Fora Formation where several hominin footprints are preserved along with those of other animals such as hippopotamus [2]. At the second site, footprints from six individuals have been discovered 70 km to the north at Ileret [7], [14]. Here a series of hominin footprints preserve anatomical details consistent with a forward-pointing large toe and clearly distinguished lateral arch. The footprints are larger than those at Laetoli, and suggest that by 1.5 My humans had developed an essentially modern walking gait and are argued to have reached a similar stature to modern humans [15].

Footprints from the Middle and Upper Pleistocene are equally rare. Three hominin footprint tracks have been preserved in volcanic tuff in the Roccamonfina area of southern Italy dating to ca. 350 ky informing about gait and stride length on steep slopes [4]. In South Africa the sites of Langebaan and Nahoon date to the last interglacial (ca. 125 ky) [3], [6]. At both sites the footprints have been preserved in calcareous eolianites and provide information about height and body build at a critical time when anatomically modern humans were first emerging. Finally, at Vârtop Cave in Romania the only recorded late Neanderthal footprint is preserved in calcareous mud dating to between 97 and 62 ky [5].

A number of factors contribute to the rarity of footprints in the archaeological record. The preservation of a footprint requires the combination of soft sediments to allow an imprint to be made, a low-energy environment in which minimal erosion of the imprinted surface takes place and rapid burial of the surface by sediments deposited in a low-energy setting, such as still to slow-flowing water or by air-fall deposition. A further consideration is the subsequent exposure of the footprint surface with minimal erosion of the features. Holocene coastal and estuarine environments are locations that seem to be favourable for footprint preservation, with good examples in the UK at Sefton and in the Severn estuary where footprint surfaces have been studied over the last 20 years [16]–[22].

**Figure 2:**
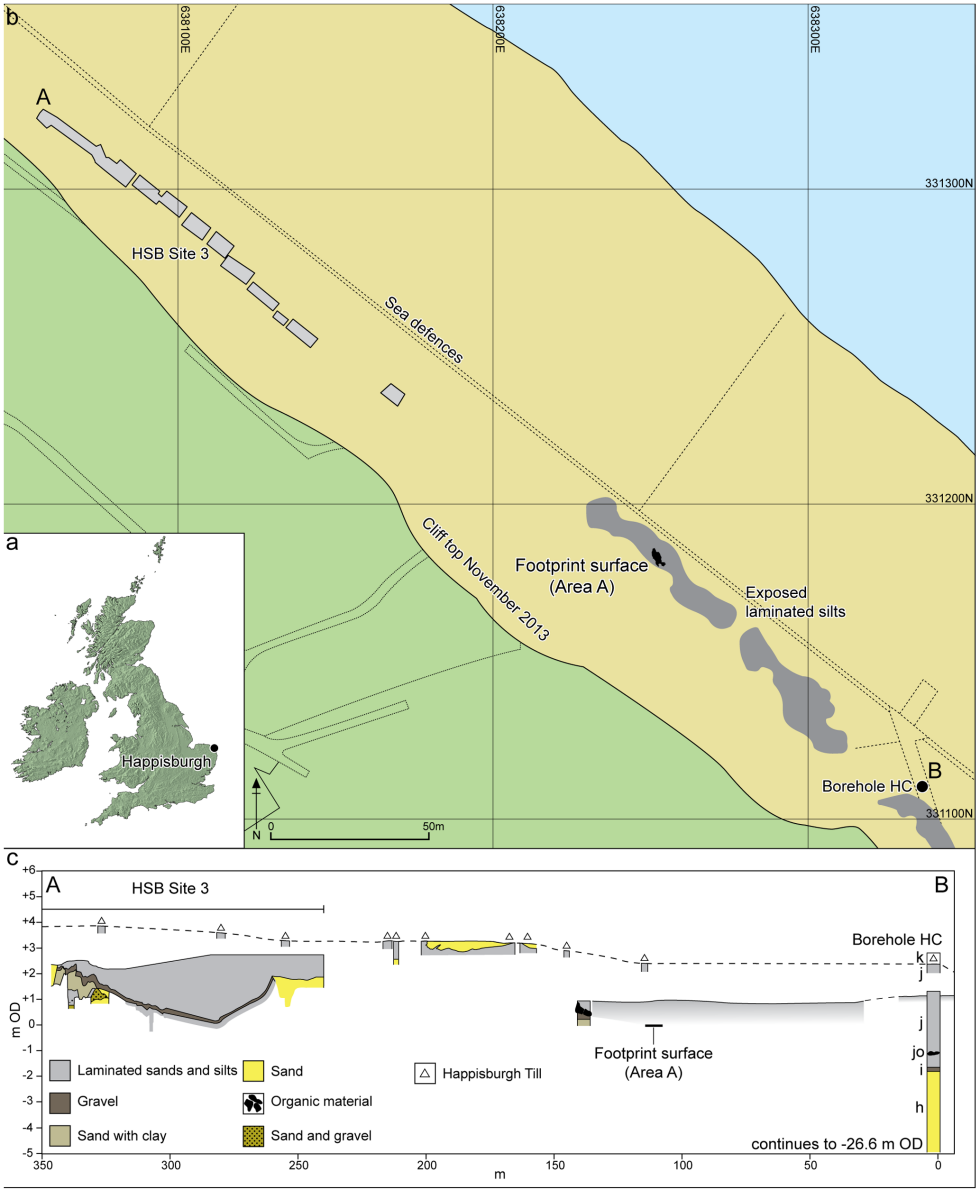
Location of Happisburgh. a. Map of UK showing location of Happisburgh; b. Plan of Happisburgh Site 3, exposed and recorded foreshore sediments, location of footprint surface and of borehole HC; c. Schematic cross-section of recorded sediments from Happisburgh Site 3 through to borehole HC showing stratigraphic position of footprint surface. Beds h–k are shown for borehole HC as recorded by West [23].

Here we report on a footprint surface found in Early Pleistocene estuarine muds at Happisburgh, UK, where preservation is due to very similar processes to those of the Holocene sites (Figure 2a). At Happisburgh the footprint surfaces have been revealed because of coastal erosion of overlying cliffs. The estuarine sediments at Happisburgh are part of the Hill House Formation (HHF) and are Early Pleistocene in age, dating to between 1 and 0.78 My. They preserve indirect anatomical evidence of the first hominins in northern Europe.

## Methods and Analysis

### Geological, environmental and archaeological context

The Early to early Middle Pleistocene succession in East Anglia is characterised by a series of estuarine, fluvial and alluvial sediments (Cromer Forest-bed Formation – CF-bF) interdigitating with near-shore marine sediments on the margins of the Crag Basin [23]. This succession dates from ca. 2 to 0.5 My and is overlain by glacial sediments, including tills of the Lowestoft and Happisburgh Formations that were laid down during Marine Isotope Stage 12 (MIS 12; ca. 450 ky). The CF-bF includes a number of important interglacial sites [23]–[26] famous for Early and early Middle Pleistocene fossil remains. It is only recently that Lower Palaeolithic archaeology has been found within the CF-bF, in particular at Pakefield, dating to ca. 700 ky [27] and at Happisburgh Site 3 (HSB3), dating to ca. 850 ky or possibly ca. 950 ky (Figure 2b and c) [28]. This evidence has extended the record of human occupation of northern Europe by at least 350,000 years and has also provided important insights into the environments of the early human occupation in northern latitudes [29]–[30].

The pre-glacial Pleistocene succession at Happisburgh was first investigated by Reid [31] and more recently by West [23]. West described the sediments exposed at the base of the cliffs and in the foreshore at a number of locations and also in a borehole near the former slipway on to the beach (Figure 2b and c). This borehole (HC) demonstrated a succession of laminated silts beneath the Happisburgh Till. Palynological data from the laminated silts indicated an interglacial vegetational succession spanning pollen zones I–IV, which West correlated with the Early Pleistocene Pastonian Stage. These sediments form a mappable unit and have been assigned to the HHF [28]. At HSB3 they consist of a series of predominantly estuarine sands and silts which infill channels, the lower bounding surfaces of which are associated with lag gravel deposits up to 0.2 m in thickness. An artefact assemblage has been recovered from these lag gravels, consisting of flint flakes, flake tools and cores. The sediments also contain a rich assemblage of fauna and flora which suggest that the archaeological evidence can be attributed to the later part of an interglacial. This interglacial is dated on the basis of biostratigraphical and palaeomagnetic evidence to the latter part of the Early Pleistocene, perhaps MIS 21 or MIS 25 [28]. The similarities in the sedimentology and palynology of the sediments at HSB3 with the laminated silts in borehole HC suggest that they are part of the same complex of channel fills; in addition, the laminated sediments can be traced laterally between the two locations though the exact stratigraphic relationship between the channel infills remains uncertain (Figure 2c).

### The footprint surface

Over the last two years, continued erosion of the cliffs, combined with particularly severe scouring and removal of the modern beach deposits during winter storms, has revealed new exposures of the HHF. The exposures are located between HSB3 and HC (Figure 2b and c) and when beach scour was extensive, horizontal surfaces of laminated silts could be traced laterally from the vicinity of HC north westwards for 150 m towards HSB3. Therefore continuity of these sediments with the laminated silts in borehole HC can be demonstrated.

**Figure 3:**
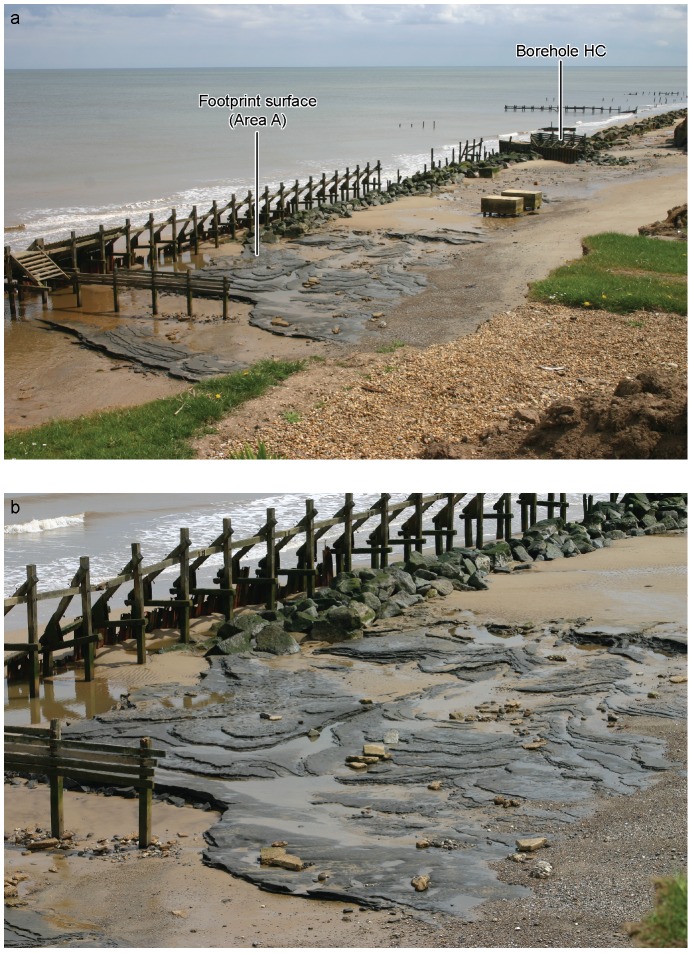
Photographs of Area A at Happisburgh. a. View of Area A and borehole HC from cliff top looking south. b. View of Area A from cliff top looking south. Photos: Martin Bates.

**Figure 4:**
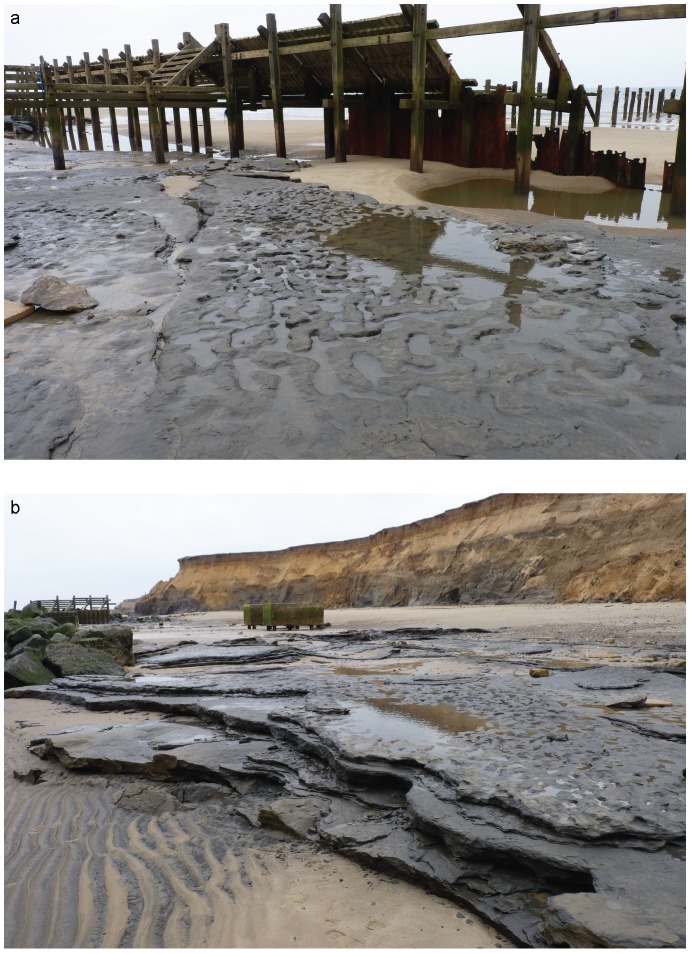
Photographs of Area A at Happisburgh. a. View of footprint surface looking north. b. View of footprint surface looking south, also showing underlying horizontally bedded laminated silts. Photos: Simon Parfitt.

**Figure 5:**
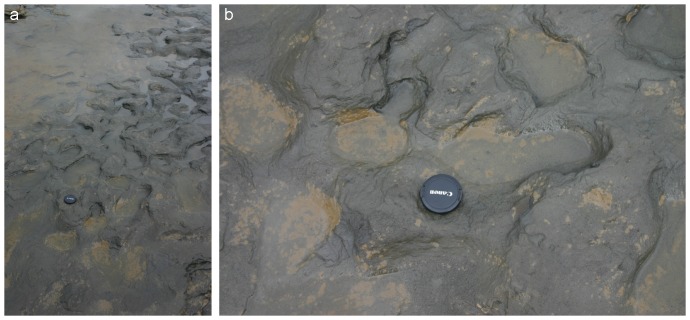
Photographs of Area A at Happisburgh. a. Footprint surface looking north-east. b. Detail of footprint surface. Photos: Martin Bates.

In early May 2013 an area of laminated silts was exposed (Area A) approximately 100 m north west of the location of borehole HC (Figures 3–5). At this site removal of beach sand had exposed the laminated silts to wave erosion; the bedding surfaces provided natural planes of weakness and the washing out of sandy laminae resulted in the removal of layers of laminated sediments and the exposure of undisturbed bedding surfaces (Figures 3–4). In most cases these surfaces are flat or gently undulating and display ripple structures formed during the original deposition of the sediments (see below). However, one horizon had very different surface characteristics where a series of hollows ranging from circular to elongate in outline were visible over an area of ca. 12 m^2^ (Figures 4–5). The elongate hollows were generally 30–50 mm in depth, 140–250 mm in length and 60–110 mm in width. The visual similarity to Holocene footprint surfaces prompted more detailed investigation of this horizon. However, as the surface was located in the inter-tidal zone of a rapidly eroding coastline and was therefore prone to rapid destruction by wave action or to reburial as the beach was re-established, it presented particular challenges for recording and analysis of the features. Over the following two weeks the surface was recorded using multi-image photogrammetry (MIP) and laser-scanning techniques. However, the features became less distinct as a result of erosion over successive tidal cycles and they had been completely removed by the end of May 2013.

The combination of tides, encroaching beach sand, weather conditions and time constraints made recording the surface extremely difficult. Prior to recording, water was used to wash away the beach sand that had been deposited during previous high tides, though it was impossible to completely clear the surface and remove all water from the hollows due to persistent rain. Field-measurement of the hollows was not possible because of the time constraints, but MIP proved to be an effective method for rapid recording of the surface features and allowed subsequent metric analysis. This method uses digital photographs taken from multiple positions around and above a subject, which are then processed to produce a 3D record of the surface (see Information S1). Laser-scanning of the surface was attempted on a subsequent visit, though the progressive deterioration of the surface morphology resulted in the features being very poorly defined.

North Norfolk District Council gave permission to work on the foreshore. No archaeological permits or licenses were required as the site is not listed under the ‘Ancient Monuments and Archaeological Areas Act 1979’ (http://www.legislation.gov.uk/ukpga/1979/46).

## Results

Extensive exposures of the laminated silts have been observed and recorded at Happisburgh since 2005 and have typically exhibited surfaces and features consistent with the range of processes found in an estuarine environment. Such environments can produce ripples and other bedforms formed by waves or current action, characterised by parallel, sometimes bifurcating crests, symmetrical or asymmetrical cross profiles and sharp ridge crests [32]–[33]. A range of marks may also be formed as the result of either stationary or moving objects on a sediment surface, which can create a variety of features [32]. None of these depositional features or surface marks is visible in Area A, though ripples were commonly observed on adjacent exposed surfaces. The marked dissimilarity of Area A to adjacent areas of laminated silts (Figure 4a) suggests that these features are not the product of normal depositional or erosional processes within an estuarine environment.

A recent origin for these features from human or animal activity can be excluded as the exposed sediments are compacted, have low moisture content and are therefore too firm to preserve recent imprints. Given the similarity of the hollows observed in Area A to Holocene footprint surfaces, the most likely explanation is that the majority of hollows can be interpreted as ancient footprints.

**Figure 6:**
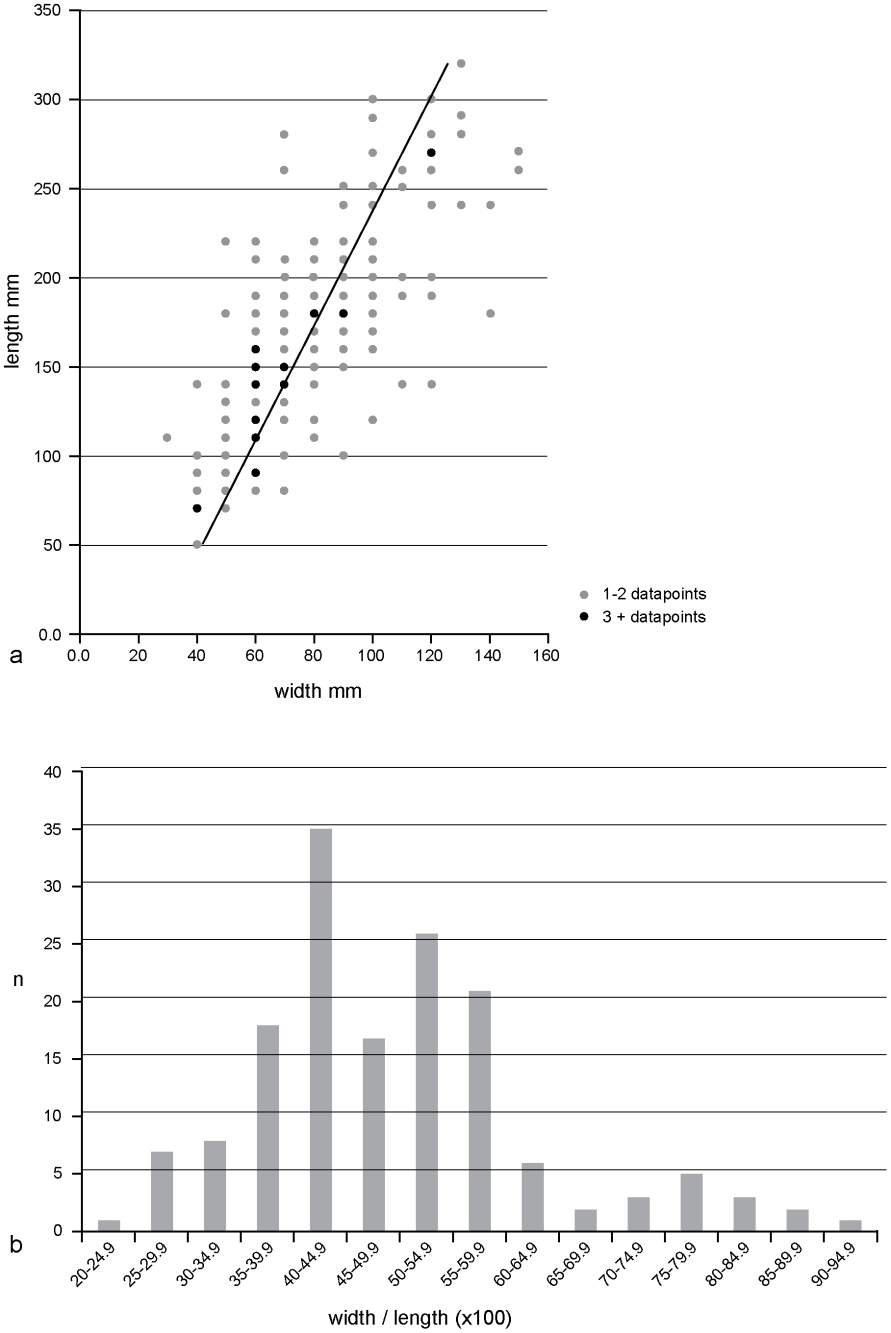
Measurements of the surface hollows in Area A at Happisburgh. a. Plot of length and width measurements of 155 hollows on recorded surface with line of regression; b. Histogram of 155 recorded hollows showing width/length ×100.

To test this hypothesis, the surface was analysed using vertical images produced from the MIP. Depth measurements were not possible as water or sand was often retained in the base of the prints and therefore the verification tests used by Morse et al. could not be adopted [34]. The initial analysis considered all the visible hollows on the surface by taking maximum length and width measurements. A total of 152 hollows were measured and this revealed that the lengths and widths have means and standard deviations of 172±60 and 80±27 mm respectively. The width/length scatter diagram clearly shows a preponderance of elongated features on this surface (correlation coefficient (*r*) = 0.73; Figure 6a) and the width/length ratio (x100) histogram has a mode of 40–44.9 (Figure 6b). The majority of hollows therefore show dimensions within the expected range of juvenile and adult hominin footprints (see below).

**Figure 7:**
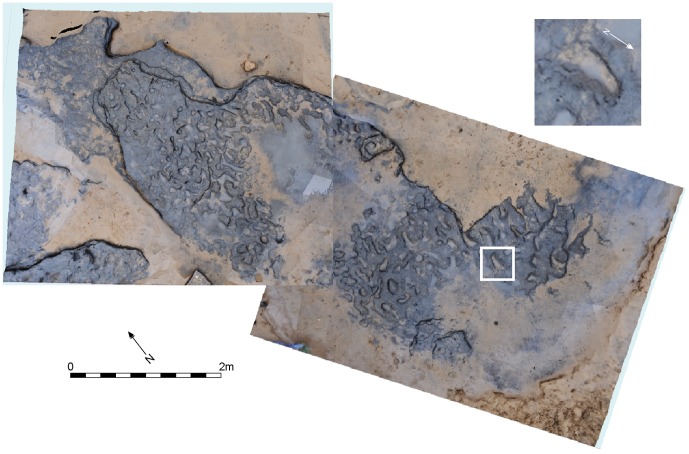
Vertical image of Area A at Happisburgh with model of footprint surface produced from photogrammetric survey with enlarged photo of footprint 8 showing toe impressions.

**Figure 8:**
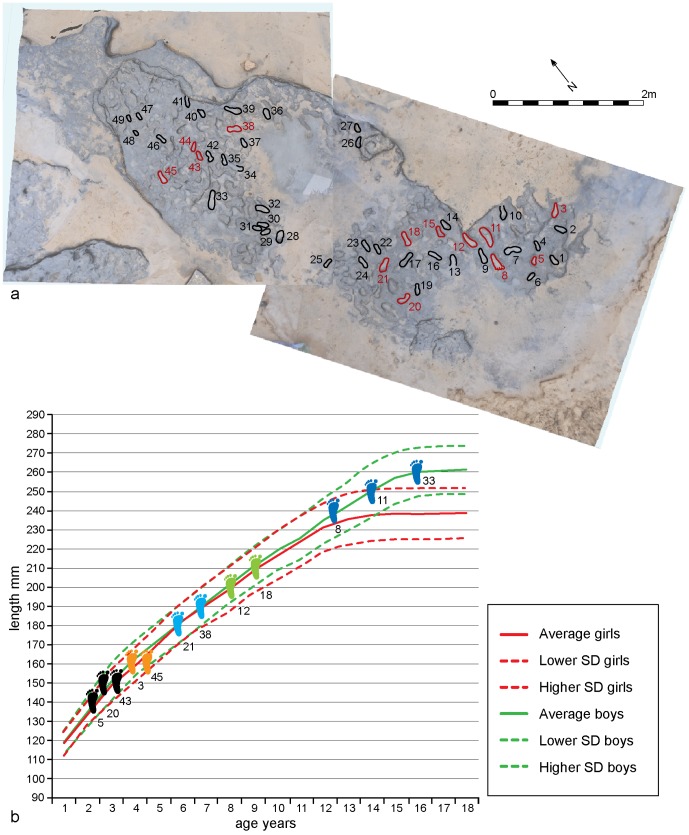
Vertical image of Area A at Happisburgh. a. Model of footprint surface generated from photogrammetric survey showing the 12 prints used in the metrical analyses of footprint size; b. Plot of length and width measurements of 12 prints showing possible individuals. Means and standard deviations for foot length and age for modern populations are also shown.

It is therefore argued that the shape of the footprints suggests that they were most likely to have been formed by hominins and none of the prints are consistent with those formed by other mammals [18]. In some cases, left or right and front or back of the foot were also apparent, including one instance of toes, provided information about direction of movement (Figure 7–8). The depth of the imprints is consistent with formation in a soft-stiff muddy substrate, as firm mud does not retain footprint impressions and semi-liquid mud has insufficient strength to retain a clear, undeformed impression [18]. The less elongated features might also be hominin footprints, where impressions from just heels or the front of feet have been preserved, or overprinting has obscured original features. The time elapsed from initial exposure to recording will also have led to some erosion of the surface, which will have affected the shape and clarity of the prints.

Quantitative analysis of footprint dimensions was limited to 12 prints where complete outlines could be clearly identified for accurate measurement of length and width (Figure 8). Print lengths vary from ca. 140 to 260 mm, indicating that they were made by several people of different ages. Taking into account slippage and erosion of the footprints, the lengths possibly indicate five individuals. The foot index (foot width:foot length ×100) has a range of 33 to 50 (mean = 39).

Stature can be estimated from foot length. Estimates from various recent populations, including adults, juveniles and both sexes produce a mean ratio of 0.15 for foot length:stature [15], [35]–[40]. Based on skeletal evidence it is also thought that the body proportions of Middle Pleistocene hominins was similar to modern humans [41]–[42] (see Information S1). Although there will be variation between foot and footprint length [15], stature estimates using the 0.15 ratio indicate a height range between 0.93 and 1.73 m, suggesting the presence of adults and children (Table 1).

**Table 1:** Length and breadth measurements of 12 footprints from Happisburgh showing estimated foot area, stature, body mass (from footprint area), body mass (from footprint length) and foot index (width:length ratio ×100).

Print	Footprint length (mm)	Footprint breadth (mm)	Footprint area (mm^2^)	Stature (m)	Body mass (kg) from footprint area	Body mass (kg) from footprint length	Foot index
3	160	80	12800	1.07			50
5	140	60	8400	0.93			43
8	240	110	26400	1.60	53	48	46
11	250	90	22500	1.67	48	50	36
12	200	90	18000	1.33			45
18	210	80	16800	1.40			38
20	150	50	7500	1.00			33
21	180	70	12600	1.20			39
33	260	90	23400	1.73	49	52	35
38	190	70	13300	1.27			37
43	150	50	7500	1.00			33
45	160	60	9600	1.07			38

The regression used from footprint area is Mass = 23.61+(0.11 × footprint area) [15]. The regression used from footprint length is Mass = 4.71+(1.82× footprint area) [15].

Body mass estimates have been obtained from footprint area using a regression based on the Daasenach experimental dataset [14]. As the muddy substrate at Happisburgh would probably have made running difficult the ‘walk-only’ regression was employed. The experimental dataset involved just adults, so only the three prints >230 mm in length were used. Estimated body masses range from ca. 48 to 53 kg using the regression based on footprint area or ca. 48 to 52 kg using the regression based on footprint length. However, as the experiments also showed considerable variation around the footprint area:body mass regression line, the estimates of body mass at Happisburgh should be treated with caution [15].

**Figure 9:**
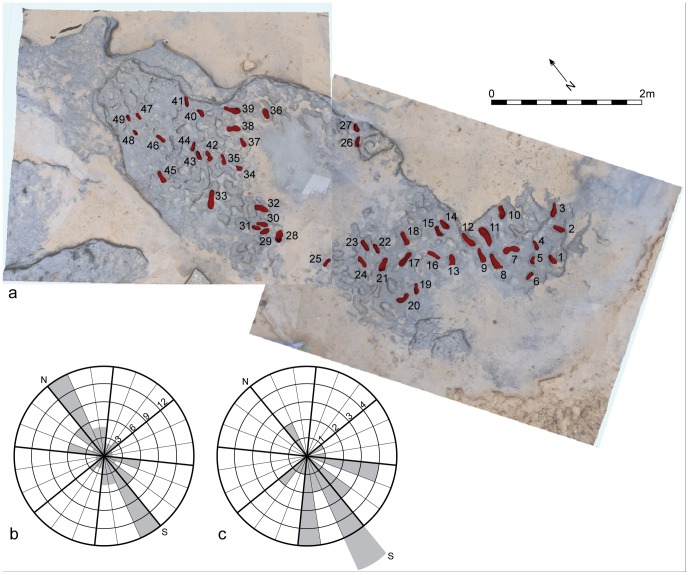
Vertical image of Area A at Happisburgh. a. Model of footprint surface produced from photogrammetric survey showing the prints used in the analyses of footprint orientation and direction; b. Rose diagram showing orientation data for 49 prints; c. Rose diagram showing direction of movement for 29 prints.

**Figure 10:**
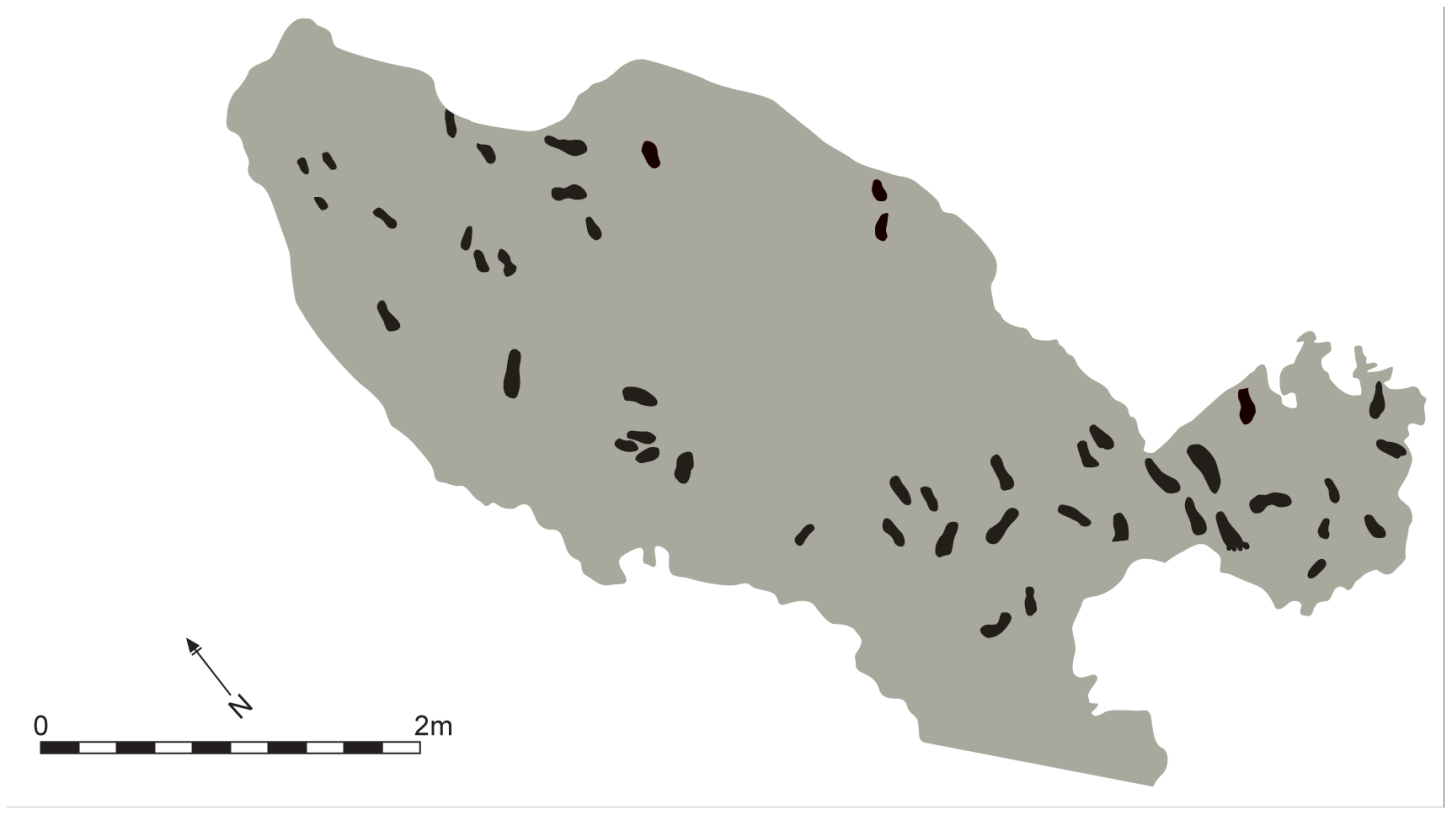
Area A showing the recorded surface area with footprints highlighted.

For the orientation studies a larger dataset of 49 prints were analysed (Figures 9–10). From this dataset a distinct preferred south-north orientation can be detected (Figure 9b; Table 2). In 29 cases where the arch and the front/back of the foot can be identified, the direction of movement can also be assessed, showing a preferred direction of movement to the south (Figure 9c; Table 2).

**Table 2:** Orientation, direction and possible left or right feet for 49 prints from Happisburgh. Degrees are given from north.

Footprint	Orientation	Direction	Interpretation left/right foot
1	176/376	176	
2	142/322	142	
3	44/224	224	Left
4	12/192	192	Left
5	44/224	224	Right
6	79/259		
7	117/297		
8	10/190	190	Right
9	9/189	189	Right
10	26/206	206	Left
11	0/180	0	Right
12	175/355	175	Right
13	29/209		
14	175/355	175	
15	6/186	186	Right
16	157/337		
17	75/255	255	
18	7/187		
19	33/213	213	Right
20	81/261	261	Left
21	62/242	242	Left
22	8/188		
23	6/186		
24	177/357		
25	74/254		
26	41/221	221	Right
27	12/192	192	Right
28	50/230		
29	93/273	93	
30	138/318	138	
31	138/318	138	Right
32	142/322		
33	40/220	220	Left
34	149/329		
35	22/202	202	Left
36	11/191	11	
37	11/191	191	
38	121/301	121	Left
39	139/319	139	Right
40	174/354		
41	27/207		
42	9/189		Left
43	16/196		Left
44	50/230	230	
45	4/184	184	Right
46	164/344		
47	0/180		
48	170/350		
49	9/189		

## Discussion

The footprint surface and the human activity that it represents can be placed within the context of the landscape at Happisburgh. The humans of mixed ages were moving in a southerly direction across the mudflats of a tidally influenced river within the upper reaches of its estuary. The mudflats were rapidly buried by tidal processes with further silts. From palynological analysis of adjoining sediments, the local vegetation consisted of a mosaic of open coniferous forest of pine (*Pinus*), spruce (*Picea*), with some birch (*Betula*). Alder (*Alnus*) was growing in wetter areas and there were patches of heath and grassland (See Information S1). This vegetation is characteristic of the cooler climate typically found at the beginning or end of an interglacial or during an interstadial period, and is consistent with genera previously identified from HC and HSB3.

The Happisburgh footprints provide the first indication of the body size of the earliest humans in northern Europe within a broader Pleistocene context. The known hominins in Europe during the Early and Middle Pleistocene were *Homo antecessor* from Atapuerca [43]–[44], *H. heidelbergensis* (e.g. Mauer, Boxgrove) or early *H. neanderthalensis* (e.g. Sima de los Huesos, Swanscombe) [45].

The evidence from Happisburgh suggests statures as large as ca. 1.73 m. As nine of the footprints indicate statures below 1.4 m, only three of the measured footprints might be considered as adults (1.60, 1.63 and 1.73 m). The adult stature estimates fall within the range of *Homo antecessor* [41] but also of *Homo heidelbergensis* and early and late Neanderthals [42]. Stature estimates based on the tali of *Homo antecessor* [41] show a mean stature of 1.73 m for male individuals and 1.68 m for female individuals. Recent stature estimates based on three samples of long bones from Sima de los Huesos, from other Neanderthal fossils and from early anatomically modern humans show mean values of 1.62, 1.61 and 1.78 m respectively [42]. The stature estimate of 1.73 m from the largest Happisburgh footprint might therefore possibly indicate a male.

The foot index (mean = 39) for the Happisburgh individuals can be compared with other past and present populations. The index is similar to Native Americans (index = 39.61) [37] and Akiak Inuit (index = 38.260) [38], but narrower than those reported for modern humans from Mexico (index adults = 44.76, juveniles = 49.58) [46], the Vârtop Neanderthal (index = 48.18) [5] and the Middle Pleistocene footprints from Italy (index = 50) [4]. The Happisburgh footprints are slightly wider than the Kenyan footprints made by *Homo erectus* or *Paranthropus boisei* (index = 36.59) [15]. Overall the estimated foot size, foot area and stature of the Happisburgh hominins correspond with the estimates for *Homo antecessor*.

## Conclusion

Happisburgh has the earliest evidence of hominin footprints outside Africa, dating to between ca. 1 and 0.78 My with estimated body dimensions that fall within the range of the evidence from *Homo antecessor* fossils. The analyses suggest a group of at least five adults and juveniles walking along the mudflats of a large river. The rarity of such evidence is equalled only by its fragility at Happisburgh, where severe coastal erosion is both revealing and rapidly destroying sites that are of international significance. The pre-glacial succession around Happisburgh has now revealed several archaeological locations of Early Pleistocene and early Middle Pleistocene age with evidence of flint artefacts, cut-marked bones and footprints. Importantly, the sites are associated with a rich environmental record of flora and fauna allowing detailed reconstructions of the human habitats and the potential for preservation of organic artefacts. Continuing erosion of the coastline will reveal further exposures of the HHF and new sites, which promise to transform our understanding of the earliest human occupation of northern latitudes.

## Supporting Information

Information S1 **Supplementary Information is provided for methods on Multi-Image Photogrammetry, the footprint analyses and pollen analysis.**
(DOCX)

## References

[1] Leakey MD, Hay RL (1979) Pliocene footprints in the Laetoli Beds at Laetoli, northern Tanzania. Nature 278: 317–323.

[2] Behrensmeyer AK, Laporte LF (1981) Footprints of a Pleistocene hominid in northern Kenya. Nature 289: 167–169.

[3] Roberts D, Berger LR (1997) Last interglacial (c. 117 kyr) human footprints from South Africa. S Afr J Sci 93: 349–350.

[4] Mietto P, Avanzini M, Rolandi G (2003) Palaeontology: Human footprints in Pleistocene volcanic ash. Nature 422: 133. 12634773 10.1038/422133a

[5] Onac BP, Viehmann I, Lundberg J, Lauritzen SE, Stringer CB, et al (2005) U–Th ages constraining the Neanderthal footprint at Vârtop Cave, Romania. Quat Sci Rev 24: 1151–1157.

[6] Roberts DL (2008) Last interglacial hominid and associated vertebrate fossil trackways in coastal eolianites, South Africa. Ichnos: An International Journal for Plant and Animal Traces 15: 190–207.

[7] Bennett MR, Harris JWK, Richmond BG, Braun DR, Mbua E, et al (2009) Early hominin foot morphology based on 1.5-million-year-old footprints from Ileret, Kenya. Science 323: 1197–1201. 19251625 10.1126/science.1168132

[8] Crompton RW, Pataky TC (2009) Stepping out. Science 323: 174–175. 10.1126/science.1170916 19251615

[9] Webb S, Cupper ML, Robins R (2006) Pleistocene human footprints from the Willandra Lakes, southeastern Australia. J Hum Evol 50: 405–413. 16343597 10.1016/j.jhevol.2005.10.002

[10] Webb S (2007) Further research of the Willandra Lakes fossil footprint site, southeastern Australia. J Hum Evol 52: 711–715. 17467039 10.1016/j.jhevol.2007.02.001

[11] Day MH, Wickens EH (1980) Laetoli Pliocene hominid footprints and bipedalism. Nature 286: 385–387.

[12] Charteris J, Wall JC, Nottrodt JW (1981) Functional reconstruction of gait from the Pliocene hominid footprints at Laetoli, northern Tanzania. Nature 290: 496–498.

[13] Deino AL (2011) 40Ar/39Ar dating of Laetoli, Tanzania. In Harrison T (editor) Paleontology and Geology of Laetoli: Human Evolution in Context. Vertebrate Paleobiology and Paleoanthropology. New York: Springer, pp. 77–97.

[14] Balter M (2013) Following the males' trail, 1.5 million years later. Science 340: 426–427. 10.1126/science.340.6131.426-b 23620031

[15] Dingwall HL, Hatala KG, Wunderlich RE, Richmond BG (2013) Hominin stature, body mass, and walking speed estimates based on 1.5 million-year-old fossil footprints at Ileret, Kenya. J Hum Evol 64: 556–568. 23522822 10.1016/j.jhevol.2013.02.004

[16] Cowell RW, Milles A, Roberts G (1993) Prehistoric footprints on Formby Point beach, Merseyside. In Middleton R (editor) North West Wetlands Survey Annual Report: 43–48.

[17] Roberts G (2009) Ephemeral subfossil mammalian, avian and hominid footprints within Flandrian sediment exposures at Formby Point, Sefton coast, north west England. Ichnos 16: 33–48.

[18] Aldhouse-Green SHR, Whittle AWR, Allen JRL, Caseldine AE, Culver SJ, et al (1993) Prehistoric human footprints from the Severn Estuary at Uskmouth and Magor Pill, Gwent, Wales. Archaeologia Cambrensis 141: 14–55.

[19] Allen JRL (1997) Subfossil mammalian tracks (Flandrian) in the Severn Estuary, S.W. Britain: mechanisms of formation, preservation and distribution. Philos Trans R Soc Lond B Biol Sci 352: 481–518.

[20] Bell MG, Caseldine A, Neumann H (2000) Prehistoric intertidal archaeology in the Welsh Severn Estuary. CBA Research Report 120. York: CBA.

[21] Allen JRL, Bell MG, Scales R (2004) Animal and human footprint-tracks in archaeology: description and significance. Archaeology in the Severn Estuary 14: 55–68.

[22] Bennett MR, Gonzalez S, Huddart D, Kirby J, Toole E (2010) Neolithic human footprints in peat from the inter-tidal zone at Kenfig, South Wales (UK). Proc Geol Assoc 121: 66–76.

[23] West RG (1980) The Pre-Glacial Pleistocene of the Norfolk and Suffolk Coasts. Cambridge: Cambridge University Press. 203 p.

[24] Preece RC, Parfitt SA, Coope GR, Penkman KEH, Ponel P, et al (2009) Biostratigraphic and aminostratigraphic constraints on the age of the Middle Pleistocene glacial succession in North Norfolk, UK. Journal of Quaternary Science 24: 557–580.

[25] Stuart AJ, Lister AM, (editors) (2010) The West Runton Elephant and its Cromerian Environment. Quat Int 228.

[26] Preece RC, Parfitt SA (2012) The Early and early Middle Pleistocene context of human occupation and lowland glaciation in Britain and northern Europe. Quat Int 271: 6–28.

[27] Parfitt SA, Barendregt RW, Breda M, Candy I, Collins MJ, et al (2005) The earliest record of human activity in northern Europe. Nature 438: 1008–1012. 16355223 10.1038/nature04227

[28] Parfitt SA, Ashton NM, Lewis SG, Abel RL, Coope GR, et al (2010) Early Pleistocene human occupation at the edge of the boreal zone in northwest Europe. Nature 466: 229–233. 20613840 10.1038/nature09117

[29] Candy I, Silva B, Lee JR (2011) Climates of the early Middle Pleistocene in Britain: Environments of the earliest humans in northern Europe. In Ashton NM, Lewis SG, Stringer CB (editors). The Ancient Human Occupation of Britain Project. Amsterdam: Elsevier, pp. 11–21.

[30] Ashton NM, Lewis SG (2012) The environmental contexts of early human occupation of north-west Europe: the British Lower Palaeolithic record. Quat Int 271: 50–64.

[31] Reid C (1882) The Geology of the Country around Cromer. London: HMSO.

[32] Reineck HE, Singh IB (1973). Depositional Sedimentary Environments. New York: Springer-Verlag.

[33] Chakrabarti I (2005) Sedimentary structures of tidal flats: A journey from coast to inner estuarine region of eastern India. Journal of Earth System Science 114: 353–368.

[34] Morse SA, Bennett MR, Gonzalez S, Huddart D (2010) Techniques for verifying human footprints: reappraisal of pre-Clovis footprints in Central Mexico. Quat Sci Rev 29: 2571–2578.

[35] Martin R (1914) Lehrbuch der Anthropologie 2. Jena: Fischer.

[36] Davenport CB (1932) The growth of the human foot. American Journal of Physical Anthropology 17: 167–211.

[37] Hrdlička A (1935) The Pueblos. With comparative data on the bulk of the tribes of the Southwest and northern Mexico. Am J Phys Anthropol 20: 235–460.

[38] Anderson M, Blais M, Green WT (1956) Growth of the normal foot during childhood and adolescence. Length of the foot and interrelations of foot, stature, and lower extremity as seen in serial records of children between 1–18 years of age. Am J Phys Anthropol 14: 287–308. 13362492 10.1002/ajpa.1330140221

[39] Robbins LM (1985) Footprints: Collection, analysis, and interpretation. Springfield: Charles C Thomas.

[40] Pales L, Garcia M, de Saint-Péreuse MT (1976) Les Empreintes de Pieds Humains dans les Cavernes. Archives de l'Institut de Paléontologie Humaine. Mémoires 36. Paris: Masson. 166 p.

[41] Pablos A, Lorenzo C, Martínez I, Bermúdez de Castro JM, Martinón-Torres M, et al (2012) New foot remains from the Gran Dolina-TD6 Early Pleistocene site (Sierra de Atapuerca, Burgos, Spain). J Hum Evol 63: 610–623. 22921478 10.1016/j.jhevol.2012.06.008

[42] Carretero JM, Rodríguez L, García-González R, Arsuaga JL, Gómez-Olivencia A, et al (2012) Stature estimation from complete long bones in the Middle Pleistocene humans from the Sima de los Huesos, Sierra de Atapuerca (Spain). J Hum Evol 62: 242–255. 22196156 10.1016/j.jhevol.2011.11.004

[43] Carbonell E, Bermudez de Castro JM, Arsuaga JL, Allue E, Bastir M, et al (2005) An Early Pleistocene Hominin Mandible from Atapuerca-TD6, Spain. Proc Natl Acad Sci USA 102: 5674–5678. 15824320 10.1073/pnas.0501841102 PMC556125

[44] Carbonell E, Bermúdez de Castro JM, Parés JM, Pérez-González A, Cuenca-Bescós G, et al (2008) The first hominin of Europe. Nature 452: 465–469. 18368116 10.1038/nature06815

[45] Stringer CB (2012) The status of *Homo heidelbergensis* (Schoetensack 1908). Evol Anthropol 21: 101–107. 22718477 10.1002/evan.21311

[46] González S, Huddart D, Bennett MR, González-Huesca A (2006) Human footprints in Central Mexico older than 40,000 years. Quat Sci Rev 25: 201–222.

