# Supplementary material for: Hominin Footprints from Early Pleistocene Deposits at Happisburgh, UK
Source: PLoS One. 2014 Feb 7;9(2):e88329. doi: 10.1371/journal.pone.0088329 (PMC3917592; doi:10.1371/journal.pone.0088329)
Supplement: Information S1 — Supplementary Information is provided for methods on Multi-Image Photogrammetry, the footprint analyses and pollen analysis. (DOCX) [file pone.0088329.s001.docx]

**Ashton et al** **Supplementary Information**

**Multi-image Photogrammetry**

Three dimensional reconstruction of the footprints was produced from a multi-image data set collected in one phase of recording. This has been successfully used on other footprint surfaces [1]. Fieldwork comprised capturing a series of digital images using an off-the-shelf camera, though the wet weather was far from ideal for recording. Photographs were systematically taken from various positions around and above the surface which was only accessible for a short time at low tide. Although the camera's focal length was controlled during the recording process, the lens was set to autofocus, proving the technique flexible and robust given the challenging weather conditions.

The software package (AgiSoft's Photoscan) used to produce the models employs a combination of computer vision algorithms such as Structure from Motion (SfM) and stereo-matching. In the first step of processing in which the photographs are aligned, the software identifies and tracks the movement of points of interest in the images. This phase results in the generation of a sparse point cloud and determination of camera position and calibration for each image. In the next phase, the software is used to build geometry using the information generated in the previous step and finally it is used to texture the resulting model using the original image set.

Due to the difficult recording conditions, Photoscan's masking feature was used in order to eliminate sections/areas of the images which may have caused the software to struggle with identifying discrete points of interest (e.g. areas with high incidence of water droplets in puddles). Processing was completed in sections (or 'chunks' as they are referred by Agisoft) and specific areas of interest such as single prints were processed separately using focused data sets.

This capture approach was selected primarily for its flexibility and potential to provide 3D visualisations of relatively complicated geometry. Furthermore, MIP incorporating Photoscan is an increasingly popular recording tool within archaeology [2-4].

**Footprint measurements**

*In situ* measurement of the footprints was not possible, so the analysis was based on the MIP vertical images. Initially 35 complete footprints were measured, but after re-measuring only those that gave consistent readings (within 10% of the first reading), 12 prints were finally measured and included in the metric analysis.

The possible number of individuals was determined by comparison of footprint length. Experimental footprints have shown mean errors of between 1.7% and 14.5% to foot length [5] therefore footprints falling within ±10% of each other were considered to have been made by the same or a similar-sized individual. This resulted in five possible clusters, perhaps representing five individuals. Footprints 5, 20 and 43 are more difficult to distinguish from footprints 3 and 45, and could be interpreted in several ways.

Stature was calculated from footprint length as an approximation of foot length [4]. A global ratio of foot length:stature was reported as 0.15 [6] and studies on various modern populations support this. Davenport reported foot length:stature ratios ranging from 0.137 in Galibi Indians to 0.16 in Bavarians [7]; the unshod Daasenach have ratios between 0.138 and 0.162 (mean = 0.15) [5]; eleven Native American populations had mean ratios of 0.149 [8]; and the cold-adapted Akiak Eskimo yielded mean male and female ratios of 0.146 and 0.145 respectively [9]. Therefore for this paper the global ratio of 0.15 has been used, although with caution given the potential errors of estimating height from footprints.

Stature comparisons with Early and Middle Pleistocene hominins are based on skeletal evidence from *Homo antecessor*, *H. heidelbergensis* and *H. neanderthalensis* using regressions calculated from stature: long bone length. Although this varies among different populations [10-12], it has been suggested that Middle Pleistocene humans from Europe had similar body proportions to modern temperate European populations [13]. Some researchers have also proposed that a global ratio provides the most accurate predictor of stature when racial affinity is unknown [14-16]. Using Euroamerican stature regressions from foot bones it has been estimated that *Homo antecessor* had male and female statures of 1.73 ± 0.07 m and 1.68 ± 0.05 m respectively, which are taller than male/female stature estimates for Neanderthals of 1.67 ± 0.06 m and 1.54 ± 0.05 m and for those from Sima de los Huesos of 1.69 ± 0.04 m and 1.58 ± 0.02 m [17-19]. The stature estimates based on the footprints at Happisburgh are consistent with those of *H. antecessor*.

Although body mass has been suggested to be higher relative to stature in populations from northern latitudes [20], recent work by Stock et al. (personal communication) suggests that this adaptation to cold is not seen in most European skeletal fossil assemblages. Because of this, the cool temperate climate and the lack of comparative footprint regressions, body mass estimates are calculated from footprint area using the ‘walk only’ regressions based on the Daasenach [5]. Given these uncertainties, the body mass estimates for Happisburgh should be treated with caution.

**Pollen Analysis**

The sediment sample used for the pollen analysis was from the same set of sediments and within 15 m of the footprint surface. The sample was prepared by standard chemical procedures, then lightly stained with safranin, dehydrated in tertiary butyl-alcohol, and the residues mounted in 2000 cs silicone oil. Slides were examined at a magnification of 400x (1000x for critical examination) by equally-spaced traverses across slides to reduce the possible effects of differential dispersal on the slides. A count of 531 land pollen and spores was made. Pollen identification, where necessary, was aided using the key of Moore *et al.* and a modern pollen reference collection [21].

The pollen assemblage is dominated by coniferous pollen: pine (*Pinus;* 48%) accounts for about half the total land pollen sum (TLP) with some spruce (*Picea;* 17%). Alder (*Alnus;* 5%) and birch (*Betula;* 6%) are also present but at lesser values. The pollen of plants characteristic of open heath and grassland, including heaths and heathers (Ericales) and grasses (Poaceae) plus a few herb taxa, make up about 20% of the TLP. The environmental picture is one of a mosaic of vegetation types with coniferous forest, probably quite open, with heathland and grassland. Some alder was growing in damper areas. Such an environment is characteristic of a cool climate as is found at the beginning or end of an interglacial or during an interstadial period.

**Supplementary Information References**

1. Hatala KG, Dingwall HL, Wunderlich RE, Richmond, BG (2013) The relationship between plantar pressure and footprint shape. J Hum Evol 65: 21-28.

2. Brutto ML, Meli P (2012) Computer Vision Tools for 3D Modelling in Archaeology. International Journal of Heritage in the Digital Era 1: 1-6.

3. Verhoeven G (2011) Taking Computer Vision Aloft: Archaeological Three-dimensional Reconstructions from Aerial Photographs with PhotoScan. Archaeol Prospect 18: 67-73.

4. Verhoeven G, Doneus M, Briese C, Vermeulen F (2012) Mapping by matching: a computer vision-based approach to fast and accurate georeferencing of archaeological aerial photographs. J Archaeol Sci 39: 2060-2070.

5. Dingwall HL, Hatala KG, Wunderlich RE, Richmond BG (2013) Hominin stature, body mass, and walking speed estimates based on 1.5 million-year-old fossil footprints at Ileret, Kenya. J Hum Evol 64: 556-568.

6. Martin R (1914) Lehrbuch der Anthropologie 2. Jena: Fischer.

7. Davenport CB (1932) The growth of the human foot. Am J Phys Anthropol  17: 167-211.

8. Hrdlička A (1935) The Pueblos. With comparative data on the bulk of the tribes of the Southwest and northern Mexico. Am J Phys Anthropol  20: 235-460.

9. Anderson M, Blais M, Green WT (1956) Growth of the normal foot during childhood and adolescence. Length of the foot and interrelations of foot, stature, and lower extremity as seen in serial records of children between 1–18 years of age. Am J Phys Anthropol  14*:* 287-308.

10. Sciulli PW, Schneider KN, Mahaney MC (1990) Stature estimation in prehistoric Native Americans of Ohio. Am J Phys Anthropol  83: 275-280.

11. Duyar I, Pelin C (2003) Body height estimation based on tibia length in different stature groups. Am J Phys Anthropol  122: 23-27.

12. Celbis O, Agritmis H (2006) Estimation of stature and determination of sex from radial and ulnar bone lengths in a Turkish corpse sample. Forensic Sci Int 158: 135-139.

13. Carretero JM, Rodríguez L, García R, Gómez A, Arsuaga JL (2005) Upper limb long bones from Sima de los Huesos site (Sierra de Atapauerca, Burgos, Spain). Am J Phys Anthropol  128: 84-84.

14. Feldesman MR, Lundy JK (1988) Stature estimates for some African Plio-Pleistocene fossil hominids. J Hum Evol 17: 583-596.

15. Feldesman MR, Kleckner JG, Lundy JK (1990) Femur/stature ratio and estimates of stature in mid‐and late‐Pleistocene fossil hominids. Am J Phys Anthropol 83: 359-372.

16. Feldesman MR, Fountain RL (1996) “Race” specificity and the femur/stature ratio. Am J Phys Anthropol  100: 207-224.

17. Pablos A, Lorenzo C, Martínez I, Bermúdez de Castro JM, Martinón-Torres M et al. (2012) New foot remains from the Gran Dolina-TD6 Early Pleistocene site (Sierra de Atapuerca, Burgos, Spain). J Hum Evol 63: 610-623.

18. Sjøvold T (1990) Estimation of stature from long bones utilizing the line of organic correlation. Hum Evol 5: 431-447.

19. Carretero JM, Rodríguez L, García-González R, Arsuaga JL, Gómez-Olivencia A et al. (2012) Stature estimation from complete long bones in the Middle Pleistocene humans from the Sima de los Huesos, Sierra de Atapuerca (Spain). J Hum Evol 62: 242-255.

20. Ruff CB, Holt BM, Niskanen M, Sladék V, Berner M et al. (2012) Stature and body mass estimation from skeletal remains in the European Holocene. Am J Phys Anthropol  148: 601-617.

21. Moore PD, Webb JA & Collinson ME (1991) Pollen Analysis. Oxford: Blackwell Scientific Publications. 216 p.
